# Too much of a good thing: Immune reconstitution inflammatory syndrome in a patient with Still's disease

**DOI:** 10.1016/j.amsu.2022.104590

**Published:** 2022-09-13

**Authors:** Nuzhat Batool, David Song, Talal Almas, Abdulla K. Alsubai, Tushar Thakur, Hebatalla Ismail, Majid Alsufyani, Sebastian Hadeed, Helen Huang, Farida Kotait, Khaled Saeed Obaid Aldhaheri, Atif Bakr Sindi, Emilie Chan, Carlos Salama

**Affiliations:** a Department of Internal Medicine, Icahn School of Medicine at Mount Sinai Elmhurst Hospital, NY, USA; b Royal College of Surgeons in Ireland, Dublin, Ireland; c National University of Ireland, Galway, Ireland; d Department of Rheumatology, Icahn School of Medicine at Mount Sinai, USA; e Department of Infectious Disease, Icahn School of Medicine at Mount Sinai, USA

## Abstract

Immune Reconstitution Inflammatory Syndrome (IRIS) is a potential complication when treating non HIV immunosuppressed patients with opportunistic infections. We present a case of a 49-year-old female with Adult-onset Still's disease on prednisone 40 mg daily who came to ED with right leg weakness and intractable headache for one week. She was diagnosed with Cryptococcus meningitis. Patient completed the induction phase of antifungal therapy and the steroids were tapered over four weeks. One month after discharge, a patient was brought in to ED, minimally responsive to verbal stimuli and had new left hemiparesis with persistent right leg weakness was noted on exam. An MRI of the brain was consistent with diffuse leptomeningeal enhancement compatible with meningoencephalitis. LP was notable for elevated opening pressure of 36cmH2O and CSF studies were negative for recurrence of cryptococcal infection. Given the timeline of patients presentation one month after discontinuation of steroids, and workup consistent with sterile meningitis, immune reconstitution inflammatory syndrome was identified as the likely diagnosis. The patient was started on 50 mg of Prednisone daily. Six weeks after presentation, the patient's mental status returned to baseline, left hemiparesis resolved, and right lower extremity strength significantly improved. Clinicians should have a high index of suspicion for CNS IRIS in patients presenting with new neurologic findings in the setting of rapid discontinuation of steroids due to infection. IRIS in HIV patients with cryptococcal meningitis is a well-established entity; the purpose of this case report is to bring attention to similar inflammatory syndrome in non-HIV patients with cryptococcal meningitis.

## Highlights

• Immune Reconstitution Inflammatory Syndrome is a potential complication when treating non HIV immunosuppressed patients with opportunistic infections.
• Clinicians should have a high index of suspicion for CNS IRIS in patients presenting with new neurologic findings in the setting of rapid discontinuation of steroids due to infection.
• IRIS in HIV patients with cryptococcal meningitis is a well-established entity; the purpose of this case report is to bring attention to similar inflammatory syndrome in non HIV patients with cryptococcal meningitis.

## 1 Introduction

Immune reconstitution Inflammatory syndrome (IRIS) is a collection of inflammatory disorders with paradoxical worsening of pre-existing infectious processes that is temporally related to recovery of the immune system [1]. First described with initiation of highly active anti-retroviral therapy (HAART) in patients infected with the human immunodeficiency virus (HIV), IRIS has been reported in patients with rheumatologic conditions upon withdrawal of corticosteroid [2] and reduction in immunosuppressive therapy in organ transplant recipients [3]. We present a case of IRIS in non-HIV patients with cryptococcal meningitis presenting as worsening mental status upon discontinuation of corticosteroids.

## 2 Case presentation

A 49-year-old woman with a past medical history of Adult-onset Still Disease presented to our hospital with right leg weakness and intractable headache for a duration of one week. Outpatient medication included prednisone 40 mg daily for three months. Steroids were first prescribed by her rheumatologist, with the dose being subsequently increased to 40 mg daily by the primary care physician due to her persistent rash. Large vessel occlusion was excluded with computed tomography (CT) angiogram of the head and neck. An echocardiogram with a bubble study was negative. Magnetic resonance imaging (MRI) of the brain was performed one day later which demonstrated multiple punctate areas of diffusion restriction including left pons as well leptomeningeal enhancement and parenchymal lesions in the left vertex concerning meningitis. A lumbar puncture (LP) was warranted to definitively diagnose meningitis. However, an LP could not be obtained until three days into hospital admission based on religious beliefs in regard to doing it on certain days of the week. Her cerebral spinal fluid (CSF) opening pressure was elevated at 29.5 cm of water. When her CSF was analyzed, it was notable for a white blood cell count of 97 cells/mm^3^, a red blood cell count of 36 cells/mm^3^, and 88% lymphocytes. CSF protein was elevated to 56 mg/dL and CSF glucose levels were within normal range of 46 mg/dL. A PCR of the CSF for cryptococcus neoformans was positive and cryptococcal antigen titers were elevated to 1:8192. Yeasts were identified using India Ink staining and CSF cultures grew cryptococcus neoformans. A CSF flow cytometry was conducted, which was non-contributory to a haemato-proliferative disease. The remainder of the infectious workup included but were not limited to CSF Herpes Simplex Virus 1 and 2, Varicella Zoster Virus, Epstein-Barr Virus, Cytomegalovirus, and the Venereal Disease Research Laboratory tests, all of which was negative. A hypercoagulability workup was unremarkable.

Based on these findings, the patient was started on amphotericin B and flucytosine. However, the patient developed chills to amphotericin infusion. Flucytosine was held after one week due to pancytopenia with her platelet count dropping to 58 cells//mm^3^ and warranted a switch to fluconazole. Due to worsening transaminitis, fluconazole was stopped 12 days after initial administration. The patient completed the induction therapy with a 6-week course of amphotericin. Steroids were tapered from 20 > 10>5 > 2.5 over four weeks. She received a repeat MRI which showed improvement from her previous findings of meningitis. A serial therapeutic LP was administered for her headaches and her response to treatment was continuously monitored with CSF studies. Five weeks into treatment, the results from the CSF PCR were positive again for cryptococcus and the patient failed monotherapy with amphotericin. Her liver function tests and cell counts had normalized off fluconazole and flucytosine. Triple therapy for a second round of induction was commenced from week four to week six. She completed 6 weeks of her induction therapy in the hospital and was later discharged with oral fluconazole for eight weeks for consolidation.

**Fig. 1:**
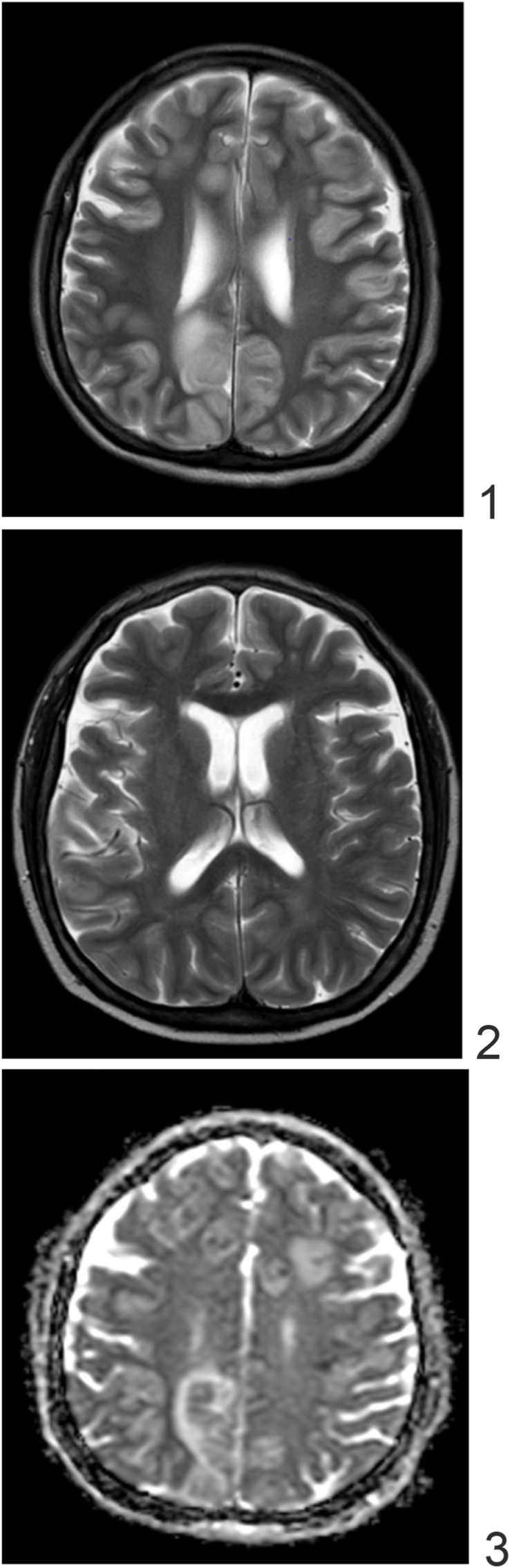
MRI brain with contrast: Figs. 1–3: Extensive abnormal T2 hyperintense signal in the cerebral cortex compatible with meningoencephalitis.

One month after discharge, the patient presented to the Emergency Department for declining mental status for a week. On examination, the patient was minimally responsive to verbal stimuli and unable to follow any commands. New left sided weakness was noted with positive bilateral Babinski signs. MRI findings showed hyperintense signals in multiple areas of cortical and subcortical enhancement consistent with meningoencephalitis (Fig. 1). LP showed 70% lymphocytes, 29% polymorphonuclear cells, CSF glucose of 40 mg/dL and CSF protein elevated to 157 mg/dL. Opening pressure was noted at 36 cm H_2_O. CSF gram stain and India Ink staining was negative. An infectious disease specialist was consulted for a possible relapse of cryptococcal meningitis. However, repeat bio fire was negative for cryptococcus and fungal cultures had no growth. The new neurologic symptoms developed precisely one month after complete cessation of steroids. Given the lack of evidence of a new infection, we attributed the worsening of her symptoms to the reactivation of the immune system after corticosteroids were withdrawn. She was started on 50 mg on prednisone as well triple therapy with amphotericin B, fluconazole and flucytosine while waiting for fungal cultures. A repeat lumbar puncture was commenced two days later and showed normalization of opening pressure to 25, improvement of CSF protein to 61 mg/dL from 158 mg/dL and CSF glucose to 68 mg/dL from 40 mg/dL. CSF PCR, India ink stains, and CSF fungal cultures remained negative for cryptococcus. Her mental status improved during hospitalization and showed motor strength recovery of her lower extremities (3+/5 on the right and 4+/5 on the left). Her dosages were tapered off from 50 mg of prednisone to 47.5 mg at one month. Upon discharge, she was prescribed 40 mg of prednisone with a slow taper of 1 mg/week as planned and 800 mg of fluconazole daily. The patient is continuously followed by our Infectious Disease and Rheumatology Department. In the most recent follow-up visit, our patient seemed to have improved and is now off steroids completely. The current paper has been reported in line with the STROCSS 2021 criteria [5].

## 3 Discussion

IRIS was first recognized in acquired immunodeficiency (AIDS) patients when in 1997 five patients developed cytomegalovirus (CMV) retinitis after initiation of HAART [[4], [5]]. There was evidence of IRIS even before that in the 1980s when the physicians observed paradoxical worsening fever, weight loss and shortness of breath after initiation of anti-tuberculosis (TB) medication with tuberculosis and leprosy [6,7]. Recent studies also suggest that IRIS can be a complication of adult-onset Still's Disease, suggesting that this inflammatory condition is T-cell driven. Given that immune dysregulation is a feature of HIV infection and attenuated by the initiation of HAART, around 10–40% of patients experienced quick deteriorations that led to IRIS due to the imbalance of cytokines and T-cells [8].

Cryptococcal meningitis accounted for 20–25% AIDS related deaths in Africa [9]. Timing of initiation of HAART in patients diagnosed with cryptococcal meningitis was studied in the COAT trial. Early initiation of HAART in patients with cryptococcal meningitis (within 1–2 weeks) was associated with 45% 26 weeks mortality compared to 30% with late initiation of HAART (5 weeks after diagnosis). Theoretically early initiation of HAART is consistent with more risk of developing IRIS. Clinically, it remained unclear whether the excess deaths in the earlier HAART group was secondary to cryptococcal meningitis vs IRIS [9,10]. Corticosteroids have remained the mainstay of therapy for IRIS associated with cryptococcal meningitis. To clarify, corticosteroids are used in treatment for IRIS not for primary prevention. Use of corticosteroids to reduce the incidence of cryptococcal IRIS in HIV patients was proven to be ineffective; and it was associated with more adverse effects and disability compared to placebo [9]. More options including thalidomide in appropriate populations are being looked into for treatment of cryptococcal IRIS in HIV patients.

IRIS in the setting of rapid discontinuation of corticosteroids is generally managed by reintroduction of steroids and slow taper. However the approach is adapted from what our knowledge for IRIS in HIV patients. In the above mentioned case the patient developed cryptococcal meningitis in the setting of prolonged steroid use for Still disease; however later developed worsening of her symptoms upon rapid discontinuation of steroids. We managed her worsening neurologic symptoms including rapid decline in mental status and worsening right lower extremity weakness with prednisone 50 mg daily for one month. A very slow taper over months was planned for our patient considering the severity of her symptoms and she had a good outcome with this treatment regimen.

## 4 Conclusion

It is important for physicians to recognize the IRIS as a potential complication when treating non-HIV immunosuppressed patients with opportunistic infections. IRIS in HIV with cryptococcal meningitis is a well-established entity; the purpose of this case report is to bring attention to similar inflammatory syndrome in non-HIV patients with cryptococcal meningitis upon abrupt discontinuation of steroids.

## Ethical approval

NA.

## Sources of funding

NA.

## Author contribution

NB, DS: conceived the idea, designed the study, and drafted the manuscript. TA, HI, AKS: conducted comprehensive literature search, screened the studies for relevant content, and created the literature review table. SH: revised the manuscript critically and refined the literature review table. EC: drafted the discussion part of the manuscript, revised the final version of the manuscript critically based on the reviewer and editorial comments. CS: Conceived the initial study idea, diagnosed the case, and gave the final approval for publication.

## Declaration of competing interest

NA.

## Consent

Written informed consent was obtained from the patient for publication of this case report and accompanying images. A copy of the written consent is available for review by the Editor-in-Chief of this journal on request.

## Registration of research studies

1. Name of the registry: NA.

2. Unique Identifying number or registration ID: NA.

3. Hyperlink to your specific registration (must be publicly accessible and will be checked): NA.

## Guarantor

Talal Almas RCSI University of Medicine and Health Sciences 123 St. Stephen's Green Dublin 2, Ireland Talalalmas.almas@gmail.com.

## Disclosures

N/A.

## Provenance and peer review

Not commissioned, externally peer-reviewed.
